# Self-organization of silicates on different length scales exemplified by amorphous mesoporous silica and mesoporous zeolite beta using multiammonium surfactants†

**DOI:** 10.1039/d0ra03828h

**Published:** 2020-06-02

**Authors:** Maria Castro, Pit Losch, Christophe Farès, Mohamed Haouas, Francis Taulelle, Eric Breynaert, Christine Kirschhock, Woojin Park, Ryong Ryoo, Wolfgang Schmidt

**Affiliations:** Max-Planck-Institut für Kohlenforschung Germany schmidt@mpi-muelheim.mpg.de; Institut Lavoisier de Versailles, UVSQ, CNRS, Université Paris-Saclay France; Center for Surface Chemistry and Catalysis, KU Leuven Belgium; CNCR, Institute for Basic Science Republic of Korea

## Abstract

In this study the structure directing effect of a gemini-type piperidine-based multi-ammonium surfactant during hydrothermal zeolite synthesis was investigated for two cases: with and without a source of aluminum. The absence of an aluminum source led to the formation of an amorphous mesoporous MCM-48 type silica material, while the presence of aluminum guaranteed the formation of zeolite beta with a hierarchical pore system. The two opposing cases were studied in a time and temperature-dependent manner. The mobility and through space interaction of these large surfactant molecules were studied by liquid state nuclear magnetic resonance (NMR) at a temperature relevant to hydrothermal synthesis (363 K) in pure water and upon addition of an aluminum and silicon source. In the gel state, at different stages of aging and hydrothermal synthesis, low angle X-ray diffraction (XRD) and solid state magic angle spinning nuclear magnetic resonance (^1^H MAS NMR) spectrometry determined the developing order within the system. At each of these different synthesis steps the respective intermediate materials were calcined. Transmission electron microscopy then allowed closer inspection of the locally developing mesoscopic order, while N_2_ physisorption was used to follow the evolution of porosity.

## Abstract 2

Reaction gels containing piperidine-based multi-ammonium OSDA yield hierarchical nano-beta zeolite in the presence of aluminum and amorphous mesoporous silica without aluminum, illustrating the highly complex thermodynamic course of zeolite formation.

## Graphical abstract

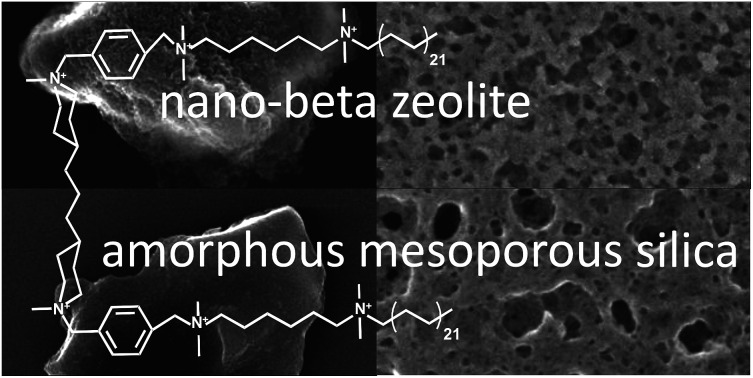

## Introduction

Hierarchical porosity is a highly desired property for zeolites.^1–3^ These microporous aluminosilicates are applied in numerous industrial processes as solid acid catalysts or in membrane technology and the selective sorption of gases.^4^ For improving mass transfer in microporous solids, introduction of complementary mesoporosity has proven highly advantageous. Mesopores in zeolite crystallites allow efficient transport of molecules from a fluid phase into the micropores of zeolite particles. The same effect can be achieved if nanoscopic zeolite particles can be assembled in a mesoscopic arrangement of the nanoparticles. Generation of hierarchical pore systems consisting of pores with increasing sizes ranging from micro- to meso- and macropores can thus be realized *via* different pathways. Destructive post-synthetic methods are based on selective extraction of specific elements from zeolite frameworks (dealumination,^5^ desilication,^6^ detitanation,^7^ or degermanation^8^). Constructive synthesis techniques on the other hand use highly sophisticated supramolecular organic structure directing agents (OSDAs).^9^ Amphiphilic, surfactant-type molecules are typically conceived. They exhibit different cationic functional groups such as quaternary ammonium or phosphonium centers^10^ to enable coulombic interaction with anionic aluminate and silicate species during zeolite crystallization under hydrothermal conditions.^11^

This route has been successfully explored by the Ryoo group for example for the syntheses of mesoporous MFI and LTA zeolites.^12,13^ Later single unit cell MFI pillared nanosheets were realized with poly-cationic OSDAs.^14^ The same group reported the synthesis of mesoporous beta zeolites using a series of novel gemini-type poly-quaternary ammonium surfactants, to which family belongs the herein studied hexa-cationic N_6_-diphe(X)_6_ (X being OH^−^, Br^−^ and/or Cl^−^; see Fig. 3 for structure).^15^ Coulomb forces, both attractive Δ*U*_attr_ and repulsive Δ*U*_rep_ (10–100 kJ mol^−1^), are driving the recognition as well as the interaction and (de-) solvation Δ*G*_solv_ between silicates, aluminates, the starting material and OSDAs. The latter may eventually mimic transition states which then can get realized in a given zeolite catalyst.^16^ The overall crystallization process of zeolites meanwhile is strongly affected by two further parameters, *i.e.*, dispersion forces and Gibbs free lattice energy. Dispersion forces Δ*U*_disp attr_ and Δ*U*_disp rep_ (<10 kJ mol^−1^) both guide the formation of an organic-inorganic non-porous hybrid material. The formation of covalent bonds (negative enthalpy) through condensation of silicates and aluminates around OSDAs (highly positive entropy due to released water) is described by the Gibbs free lattice energy Δ*G*_latt_. The latter causes zeolite crystallization processes to be generally exothermic.^17,18^ The overall free energy of a hydrothermal synthesis (Δ*G*_h.t.syn_) is defined by a complex series of transitory equilibria, challenging to follow experimentally.^19^ In sum, over time and temperature of a hydrothermal synthesis, the balance between coulombic attraction/repulsion, the dispersion attraction/repulsion forces, and finally the release of crystal lattice energy and water determine the thermodynamics (Δ*G*_h.t.syn._) of the hydrothermal OSDA-guided synthesis of microporous (alumino)-silicate materials (eqn (1)).1
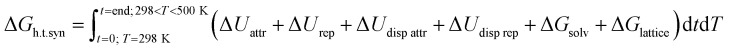


Zeolite formation mechanisms tend to be studied in colloidal precursor suspensions, enabling to follow the growth processes with non-invasive analysis techniques.^20,21^ We recently investigated the supramolecular processes governing the self-assembly between organic and inorganic species eventually leading to nano-beta zeolites in solution and in dense gels.^22^ In the first case a solution mediated or colloidal synthesis was studied with small angle X-ray scattering (SAXS) and liquid state NMR to provide complimentary insights into the system. For dense gels the study relied on MAS NMR spectroscopy. A two-step self-assembly mechanism forming first amorphous nano-objects that gradually transformed into nano-beta zeolite was proposed. The polar head group of N_6_-diphe strongly resembles 4,4′-trimethylenebis(*N*-methyl,*N*-benzyl piperidinium) (TMP^2+^), a template found to permit the synthesis of pure silica beta zeolites.^23^ The herein studied hexa-cationic N_6_-diphe(X)_6_ consists of a polar TMP^2+^ head with four additional polar alkylammonium groups and extended hydrophobic alkyl chains conferring it an amphiphilic character. Since the polar head group contained the TMP^2+^ sub-unit, we were interested in the question whether N_6_-diphe might allow the synthesis of Al-free nano-beta zeolite. Pure silica zeolites, such as silicalite-1 and all-silica beta zeolite, have been suggested as promising catalysts,^24^ and also as hydrophobic membrane materials.^25^ Some of us studied the condensation reaction between methanol and formaldehyde yielding oxymethylene ethers (OMEs) as promising diesel substitutes. This gas phase reaction was surprisingly successfully catalyzed by all-silica zeolites.^26^

We thus compared syntheses with and without aluminum in the reaction gels. We present the outcome of our study and discuss the intermediate steps leading to two very different products, namely amorphous mesoporous silica (AMS) and a hierarchically porous aluminum-containing nano-beta zeolite.

## Results and discussion

Mesoporous zeolite beta from reaction gels containing N_6_-diphe(Cl)_4_(Br)_2_ forms as aggregates of nanoscopic zeolite particles.^21,22^ The mesopores comprise smaller voids between the nano-crystallites and larger voids that are caused by phase-separation of hydrophilic and hydrophobic liquid phases during the crystallization process. Quite similar products were formed starting either from clear sols (N_6_-diphe(OH)_6_) or from rather dense gels (N_6_-diphe(Cl)_4_(Br)_2_). Thus, N_6_-diphe exhibits a strong structure directing effect favoring the formation of nano-crystallites of zeolite beta.

**Fig. 1:**
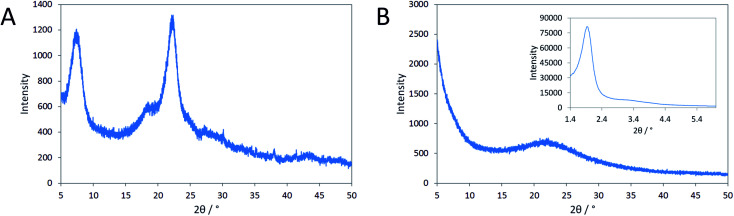
XRD patterns of (A) nano-beta obtained with aluminum after 12 h at 140 °C and (B) amorphous silica (AMS) obtained without aluminum after 14 h at 140 °C respectively. The inset shows the low-angle diffraction pattern of the AMS sample.

Here, we investigated the effect of aluminum on the formation of the solids for reaction mixtures containing N_6_-diphe(Cl)_4_(Br)_2_ as OSDA in dense gels. Reaction gels were prepared with the only difference that aluminum was present in one gel and absent in the other gel (Table S1†). As mentioned above, the polar head-group of N_6_-diphe(Cl)_4_(Br)_2_ resembles the OSDA TMP^2+^ that was used by Hould *et al.* for the synthesis of Al-free zeolite beta.^23^ Pure silica nano-beta may hence form using N_6_-diphe(Cl)_4_(Br)_2_ as the OSDA in an Al-free synthesis. Yet, wide angle XRD data clearly revealed that this is not the case (Fig. 1), nano-beta is formed if aluminum is present, whereas only amorphous silica is formed if aluminum is absent. However, low-angle XRD shows distinct reflections, indicating the presence of an ordered mesophase (inset in Fig. 1B). Inspection of the evolution of that phase with time shows that the respective mesophase has already formed during aging at room temperature and its reflections can be indexed as (211), (220), (400), and (332) reflections of a silica mesophase with cubic *Ia*3̄*d* symmetry (Fig. S1†). Apparently an MCM-48-type silica mesophase has formed at room temperature, here denoted as cAMS (cubic amorphous mesoporous silica) for simplicity. The mesostructure does not change much during aging at 60 °C, but significant changes are observed for materials obtained after hydrothermal reaction at 140 °C (Fig. S2†). The major reflection position gets shifted towards larger diffraction angles for the samples obtained after 1 and 6 h of hydrothermal reaction. Upon thermal treatment the respective *d* values change from 4.60 nm (after stirring at room temperature) to 3.96 nm during the first hour of hydrothermal reaction. With increasing time of hydrothermal reaction, the *d* value then increases again after reaction for 6 h (4.18 nm) until almost the original value is observed (4.57 nm) after 12 h of reaction. During the hydrothermal reaction, the mesostructure gets more disordered and the intensities and positions of the low-angle XRD reflections of the product obtained after 12 h do not fit to positions expected for cubic symmetry.

Tentatively they could be assigned to a disordered hexagonal symmetry (MCM-41-like, see Fig. S2†). The very broad low angle reflections and the low intensities of the smaller reflections indicate a very poorly ordered material (Fig. 1B), denoted here as AMS (amorphous mesoporous silica) for simplicity. It is noteworthy that in the Al-containing synthesis also a cubic mesophase with *Ia*3̄*d* symmetry is observed after addition of N_6_-diphe(Cl)_4_(Br)_2_ and NaOH to water at room temperature as we have shown in our previous work.^22^ That mesophase is maintained when NaAlO_2_ is added to the solution. However, upon addition of TEOS to that reaction mixture, the mesophase gets altered and upon hydrothermal synthesis is completely lost in favor of nano-beta zeolite.^22^

**Fig. 2:**
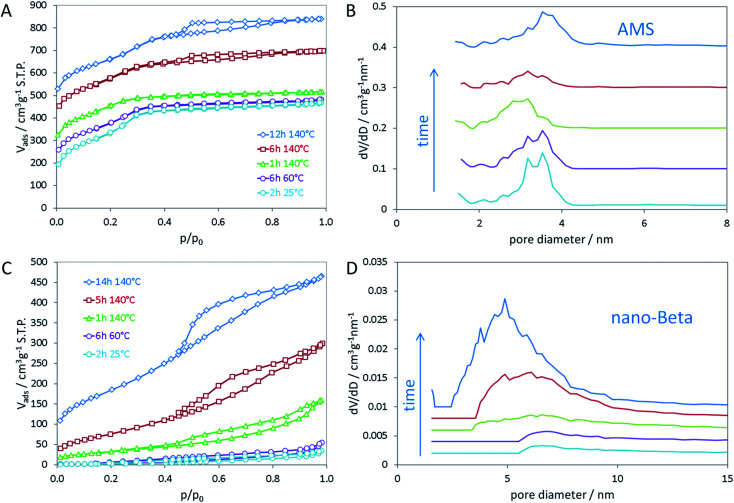
Nitrogen sorption isotherms of (A) AMS and (C) nano-beta at different stages in time and temperature of the hydrothermal synthesis; non-local density functional theory (NLDFT) was applied to calculate pore size distributions for (B) AMS and (D) nano-beta.

In the opposing case, the absence of an aluminum source led to the same mesoscopic phase with *Ia*3̄*d* symmetry, which is maintained after addition of the silica source. In a similar manner as in the synthesis of MCM-48, the silica interacts with the polar head groups of the amphiphilic OSDA molecules. Condensation of the silica then results in the formation of amorphous silica with mesoscopic order. In that manner, the data suggest that first cAMS is formed during aging at lower temperature (25 and 60 °C) which is then successively transformed into AMS during the hydrothermal reaction. This transformation is observed by shifting reflections in the low-angle XRD data. The transformation from cAMS to AMS is also noticeable from nitrogen sorption data as illustrated in Fig. 2A and B. Textural parameters of all materials investigated here are summarized in Table S1.†

The mean mesopore size of the cAMS obtained at room temperature (2 h) is 3.4 nm (Fig. 2B). During the transformation into the AMS phase, the average mesopore diameter decreases to 3.0 nm in the calcined material obtained after 1 h at 140 °C. With increasing reaction time at 140 °C and full transformation into AMS, the pore diameter increases again to 3.6 nm for the material obtained after 14 h. The described transformations agree very well with the above discussed XRD results. All cAMS and AMS materials show very high specific surface areas between 900 and 1200 m^2^ g^−1^ and high total pore volumes between 0.5 and 0.7 cm^3^ g^−1^. Since the boundary conditions for the BET algorithm are not valid for microporous materials, the specific surface areas determined for these silicas are considered apparent specific surface areas. Interestingly, the AMS materials also show microporosity to significant extent. A reason for the presence of micropores could be strong interaction of the polar head groups of the N_6_-diphe molecule with the silica. Partial embedding of the molecules, especially of the head groups, within the silica could result in the formation of micropores upon combustion of the organics during calcination. Intriguingly, the formation of microporosity indicating a strong interaction between the polar head of N_6_-diphe and silica precursors could be observed but no zeolite phase was formed.

In contrast to the Al-free synthesis, the Al-containing samples obtained at early stages of the synthesis are quite dense, as indicated by the absence of significant porosity and low specific surface areas. Only upon hydrothermal treatment porosity is generated as can be seen in Fig. 2C and D. The final nano-beta has relatively high specific surface area and pore volume as expected for the zeolite with hierarchical pore system (Table S1†). The mesopore diameters of the final nano-beta materials are slightly larger than those of the AMS materials. Extension of the reaction time from 14 h to 7 d does not change the XRD pattern of the nano-beta significantly (Fig. S3†) but the porosity of the final product increases slightly, especially with respect to microporosity from 0.03 cm^3^ g^−1^ after 14 h to 0.11 cm^3^ g^−1^ after 7 d at 140 °C. Total pore volume and apparent specific surface area after the complete hydrothermal synthesis are 0.865 cm^3^ g^−1^ and 849 m^2^ g^−1^ (Fig. S4†). A striking feature of the silica material obtained from the Al-free synthesis is that the mesopore diameters are quite small for an amphiphilic molecule such as N_6_-diphe. The extended hydrocarbon sidechains (C_22_H_45_ chain) are significantly longer than those of cetyltrimethylammonium bromide (C_16_H_33_ chain) used in the synthesis of MCM-48. One thus would expect larger pores than usually observed for MCM-48. The length of the hydrophobic side chain has been calculated to be about 2.5 nm.

The maximum distance of the ammonium group in the TMP^2+^ unit of the N_6_-diphe molecule to the end of the hydrophobic tail was calculated to be about 3.6 nm.^22^ A radial (or spoke-type) arrangement of the alkyl chains in cylindrical micelles is usually assumed for CTAB^27^ but seems very unlikely for the N_6_-diphe surfactant.

**Fig. 3:**
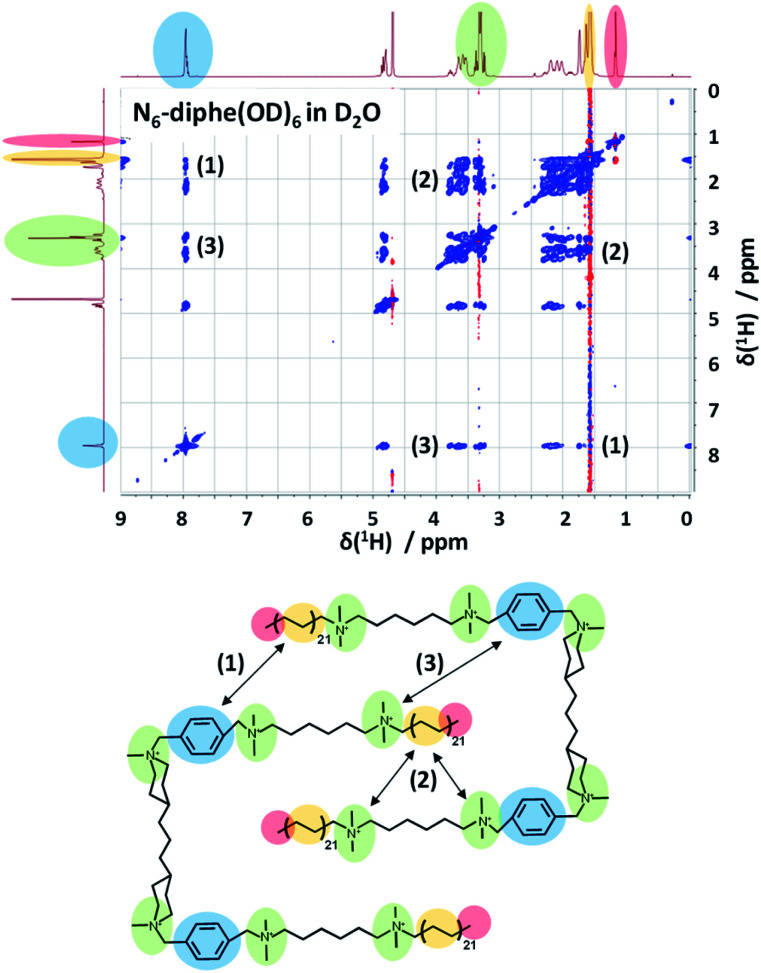
2D-NOESY spectrum of N_6_-diphe(Cl)_4_(Br)_2_ in D_2_O indicates the through space interactions of different functional groups in this large surfactant molecule. Polar groups interact with non-polar ones indicating a stacked or intercalated folding of the surfactant as represented below.

In order to gain more information on the arrangement of the OSDA, we have studied the organization of N_6_-diphe(Cl)_4_(Br)_2_ micelles in water by NMR spectroscopy. The structure and self-interaction of the pure OSDA was studied by means of 2D NOESY to extract information on through-space interactions (Fig. 3). From these analyses it can be extracted that the long C_21_H_42_ alkyl chain interact with the polar tetraalkylammonium groups as well as with the phenyl groups. This is most likely explained by a stacked or intercalated folding of the side groups on the supramolecular level as schematically indicated on the right side of Fig. 3. The observation that the long alkyl groups exhibit NOEs with tetraalkylammonium and phenyl groups shows that the chains spend a significant time located relatively close to these groups. This indicates that the disordered alkyl chains have the ability to coil up, bringing the complete chains into the vicinity of the head groups. This goes along with a reduction of their lengths by which also the micelle dimensions get reduced. Organization with ordered head-groups and disordered chains is also supported by the fact that the terminal, freely rotating methyl group only exhibits and inverted (red) single NOE to the nearest CH_2_ relative to the rest of the NOE cross-peaks (blue) of the spectrum.

NOESY typically presents positive cross-peaks for “small” molecules with fast correlation times and negative ones for “large” molecules with slow correlation times. The N_6_-diphe is a special case which shows both, meaning that the methyl groups/chains are disordered and the rest is more ordered. The organization of the N_6_-diphe(X)_6_ molecules within the micelles is thus indeed different to that of CTAB. Whereas CTAB has one alkyl chain per polar alkylammonium group, N_6_-diphe(X)_6_ only has two alkyl chains per six polar groups, most likely resulting in a less dense packing of the apolar alkyl chains. The ratio of polar groups to alkyl chains is 1 for CTAB and 3 for N_6_-diphe(X)_6_. Thus, if all polar groups tend to be located at the vicinity of the aqueous phase, significantly less alkyl chains have to organize themselves in the center of the micelles. A stacked or folded organization of the alkyl chains will then maximize the van der Waals interactions of the chains. Any such organization will result in micelles with a smaller diameter than in radial organization and consequently, the diameter of the pores in the silica will be smaller.

From the above it can be deduced that the presence of an aluminum source is essential to allow crystallization of nano-beta under the reactions conditions used for our study. From previous studies we know that the aluminum interacts strongly with the silica phase.^21,22^ Thus, it seems that the close interaction of the silica and alumina in the amorphous silica phase that forms around the organic micelles triggers the crystallization of zeolite beta during the hydrothermal reaction. The Si–O–Al bond is known to be more labile than Si–O–Si bonding, thus allowing faster exchange and engagement in distorted bonds.^28,29^ Such particular thermodynamic and kinetic features make aluminosilicate more flexible and reactive than pure silicate.

^1^H MAS NMR was used to analyze the materials after hydrothermal synthesis (Fig. S5†). In both cases a yellowish solid is obtained, magic angle spinning NMR of the protons in the OSDA can distinguish between ordered and disordered species, depending on whether sharp resolved or broad signals are observed. Nano-beta exhibits such sharp signals for the aromatic part of the N_6_-diphe molecule while the signals for the aliphatic chain are less finely resolved presumably due to dynamic movements. This is in agreement with previous conclusion that is rigid polar heads of the OSDA interact strongly with the aluminosilicate, leading to well-defined local environment and conformation.^22^ In the AMS sample on the other side, synthesized without aluminum, the same template does not appear to be immobilized in an ordered environment, neither the hydrophilic nor the hydrophobic part is leading to a defined signal in ^1^H MAS NMR. Hence, as stated above, the presence of aluminate species seems necessary to overcome the activation energy required to form crystalline domains, starting from curved micelle shapes. The micellar pre-organization was also already affected by the presence of an aluminum source. The nano-beta gel is clearly less ordered on the mesoscale, as opposed to cAMS for which an ordered self-assembled mesostructure is observed. An ordered cAMS material is already present at room temperature after mixing all the reagents. Apparently, no hydrothermal synthesis is needed to produce this material. Hydrothermal reaction only results in an apparent re-arrangement of the mesopores towards a less ordered arrangement, but does not transform this material into an all-silica micro-mesoporous beta zeolite.

**Fig. 4:**
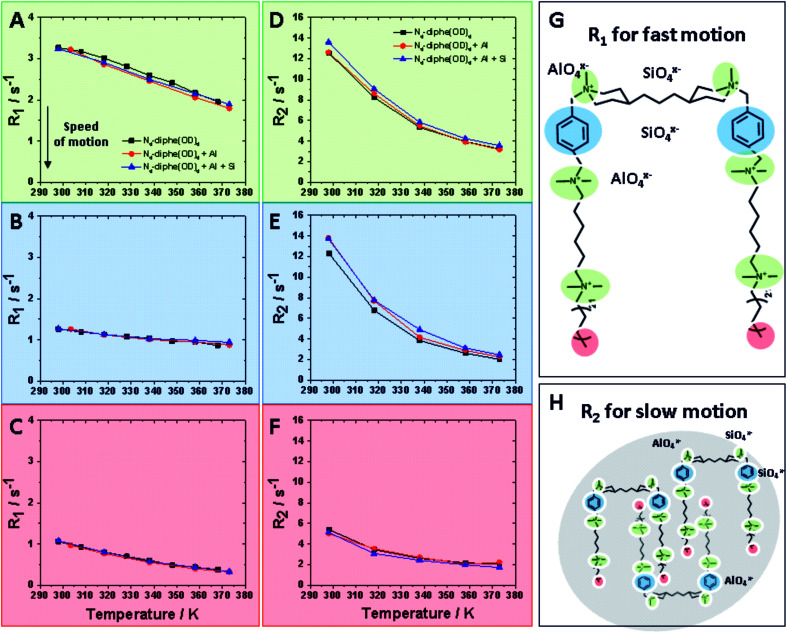
Relaxation data for N_6_-diphe from ^1^H NMR in the liquid phase at increasing temperatures: *R*_1_ and *R*_2_ data are plotted for fast microviscosity motion and slow tumbling like motion respectively. (A–C) show *R*_1_ values at temperatures ranging from room temperature to hydrothermal conditions for NR_4_^+^ group (green), aromatic group (blue) and terminal methyl group (red) respectively. (D–F) present *R*_2_ values for the same functional groups of the surfactant molecule. All these relaxation data were acquired for three different stages of the synthesis: free N_6_-diphe (black data points), N_6_-diphe interacting with an alumina source (red data points) and the complete synthesis gel with silica and alumina source (blue data points). (G and H) schematically present the measured physicochemical phenomena.

*In situ* ^1^H NMR data in the liquid phase are represented in Fig. 4. Relaxation data were recorded at different temperatures and at different stages of the synthesis. These data are interpreted qualitatively in terms of dynamic behaviour. The efficiency of relaxation of a spin magnetization is proportional to the square of its local fields, (dominated by homonuclear dipolar interactions in the case of protons (^1^H)) as well as to the random fluctuations of these interactions at key Larmor frequencies *ω* (described by density functions *J*(*ω*)). In the model of freely tumbling molecules, the relaxation rates can be expressed as follows:^30,31^

2 *R*_1_ = 1/*T*_1_ ∝ *r*^−6^(3/2)[*J*_1_(*ω*_0_) + *J*_2_(2*ω*_0_)]

3 *R*_2_ = 1/*T*_2_ ∝ *r*^−6^[(3/8)*J*_0_(0) + (15/4)*J*_1_(*ω*_0_) + (3/8)*J*_2_(2*ω*_0_)],

where *r* corresponds to dipole–dipole distance, and spectral densities for random isotropic rotation are:*J*_q_(*ω*_0_) = *C*_q_[*τ*_c_/(1 + (*ω*_0_*τ*_c_)^2^)] for *q* = 0, 1, and 2 (*C*_0_ = 24/15, *C*_1_ = 4/15, and *C*_2_ = 16/15).

*τ*_c_ is the rotational correlation time. Thus, the frequency sensitivities of *R*_1_ and *R*_2_ are defined as follows: *R*_1_ is sensitive to fast motion (*ω* ∼ *ω*_0_, 2*ω*_0_) in the ps–ns time scale (*e.g.* rotameric exchange, axial rotations, *etc.*), while *R*_2_ is sensitive to the same motions but also to much slower motions (*ω* ∼ *0*) with time scale range <μs (*e.g.* lateral diffusion in the micelles, chemical exchange, overall slow tumbling).^30^

These two different relaxations were followed and are plotted at increasing temperatures in Fig. 4. *R*_1_ values detect fast motion (Fig. 4A–C), whereas *R*_2_ values also inform on the slower motion of larger aggregates (Fig. 4D–E). In general, lower relaxation rate (*R*) values indicate faster motion; thus, unsurprisingly, mobility increases with rising temperature. Three different domains of the N_6_-diphe molecule, namely the ammonium centers NR_4_^+^ in green, the aromatic part of N_6_-diphe in blue and terminal methyl group of the aliphatic chain in red were monitored at three different stages of the synthesis. Values for free N_6_-diphe are plotted in black, N_6_-diphe interacting with an alumina source in red, and the complete synthesis gel with silica and alumina source represented in blue.

For the complex mixtures analysed in this study, no particular models can be followed. We were nonetheless interested in the *R*_2_ sensitivity to slower motion (*J*_0_(*0*)), even though both *R*_1_ and *R*_2_ are sensitive to motions on the ns time scale. In this regard, our results clearly show that the addition of Al and Si has no significant effect on *R*_1_ for all 3 spin-types (Fig. 4 and S6†). The addition of Al and Si seems to have a small effect on the signals from the polar head groups (aromatic and *N*-methyls), increasing slightly their *R*_2_ and thus seemingly slowing down the rotational correlation time. This effect is probably due to a small increase in size due to coulombic interactions, but could also be due to restricted mobility in vicinity of the inorganic species. It is reasonable to assume therefore that ionic (silico)alumina species preferentially interact with the alkylammonium centers of the molecule, thereby slightly increasing the overall size of the micelle. A soft cation [NR_4_]^+^ and a soft anion [Si_*n*_Al_*m*_O_*x*_(OH)_*y*_]^*z*−^ seem to recognize each other *via* coulombic attraction.

It can therefore be concluded that not a mere increase in the ionic strength of the liquid thanks to the addition of [Si_*n*_Al_*m*_O_*x*_(OH)_*y*_]^*z*−^ is responsible for the different outcomes in the two studied syntheses. The effect is more specific, we observed a recognition of [Si_*n*_Al_*m*_O_*x*_(OH)_*y*_]^*z*−^ by the quaternary ammonium centers of the surfactant. This selective recognition is also the likely reason why the presence of [Si_*n*_Al_*m*_O_*x*_(OH)_*y*_]^*z*−^ is required to guarantee the formation of zeolite beta. Stronger attractive Coulomb Δ*U*_attr_ and dispersion forces Δ*U*_disp attr_ may allow overcoming the activation energy towards the crystallization of zeolite beta.

In addition to this *in situ* NMR-study, we performed DOSY measurements to extract diffusion coefficients (*D*_eff_) of the surfactant at different synthesis steps (Fig. S7†). The addition of an aluminum source led to a 10–20% decrease in *D*_eff_ of the N_6_-diphe, which is part of some larger micellar structure. *D*_eff_ values drop from 2.10–2.46 10^−6^ cm^2^ s^−1^ for the free N_6_-diphe(OD)_6_ to 1.96 10^−6^ cm^2^ s^−1^ for the sample interacting with aluminate anions. From the above discussed experiments it is also apparent that the addition of alumina decreases the mobility of polar head groups of N_6_-diphe. The DOSY data hence also support the hypotheses that the whole N_6_-diphe as isolated molecule or in the micelles suffers from a reduced mobility as soon as it is interacting with an Al source.

**Fig. 5:**
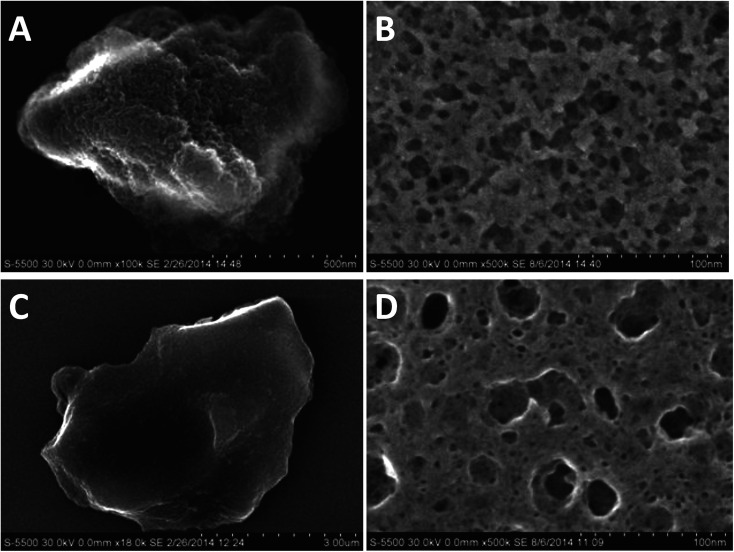
SEM micrographs (left) and microtomed cross-sections (right) of nano-beta (A and B) and AMS (C and D) respectively.

In the alkaline synthesis solution, aluminum is present as [Al(OH)_4_]^−^ anions which can readily interact with the positively charged alkylammonium groups *via* electrostatic interaction. The strong coulombic interaction then selectively decreases the mobility of the polar head group. At a longer time scale of the DOSY measurements this translates also into a reduced overall diffusion coefficient. If the silicon source (TEOS) is added next, Si(OH)_3_O^−^ monomers form upon hydrolysis of TEOS. The Si(OH)_3_O^−^ increasingly interacts with the aluminate to form an amorphous aluminosilicate species.^21,22^ The polar head groups of the N_6_-diphe molecule, which interact with the aluminate likely get incorporated into the amorphous silica that forms around the N_6_-diphe micelles. The stronger coulombic and van der Waals attractions involving the amorphous aluminosilicate and the polar head group of the N_6_-diphe molecule finally trigger the nucleation and crystallization of the nano-beta. The polar head groups get incorporated in the micropores of the nano-beta and the hydrophobic sidechains of the N_6_-diphe molecule surround the nanocrystallites, leading to a phase separation of a hydrophobic phase (containing the nanocrystallites the surfaces of which are decorated with the hydrophobic chains) and an aqueous phase. That phase separation, similar to spinoidal decomposition of oil–water mixtures, then causes the formation of the sponge-like structure of the nano-beta.^22^ The typical sponge-like arrangement of the nano-beta crystallites that forms the hierarchical pore system of that material is shown in Fig. 5B.

Interestingly, a similar sponge-like pore system is observed for the AMS material. Fig. 5 and S8† present SEM micrographs for the final materials obtained in the presence and absence of aluminum. The nano-beta (Fig. 5A) reveals its sponge-like mesoporosity when analyzing microtomed cross-sections (Fig. 5B). The AMS sample also exhibits mesoporosity as can be seen in the image of the microtomed material Fig. 5D. It presents however a less rugged and rather smooth glass-like surface at the more macroscopic level (Fig. 5C). These micrographs indicate a similar phase separation mechanism as observed for the nano-beta material leading to mesopores. The reason for this spinoidal decomposition is likely the large excess of N_6_-diphe in the reaction gels. The amount of N_6_-diphe(Br)_2_(Cl)_4_ in the gels was reckoned to form vesicular species in addition to the ordered arrangements of the cylindrical micelles. These large vesicles contain large amounts of N_6_-diphe. Finally, it is reasonable to assume that also monomolecular N_6_-diphe exists.^22^ Upon thermal treatment during the hydrothermal reaction vesicles may fuse and form lager interconnected vesicular structures, again resulting in a phase separation of polar and non-polar phases. Since the silica surrounds the rod-type micelles, it is likely that the AMS particles organize themselves in the aqueous phase rather than in the hydrophobic phase. After calcination the result is similar to that for the nano-beta material, a sponge-like hierarchical pore structure.

**Fig. 6:**
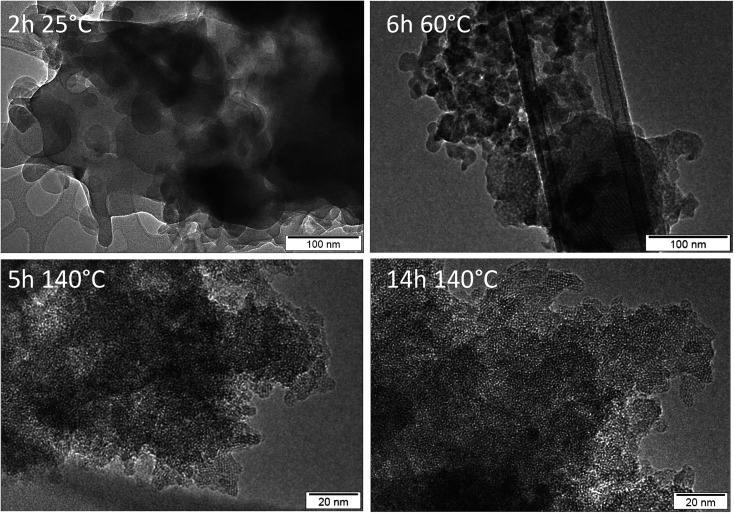
TEM images of materials after calcination of products obtained from nano-beta synthesis with aluminum as a function of synthesis time and temperature.

**Fig. 7:**
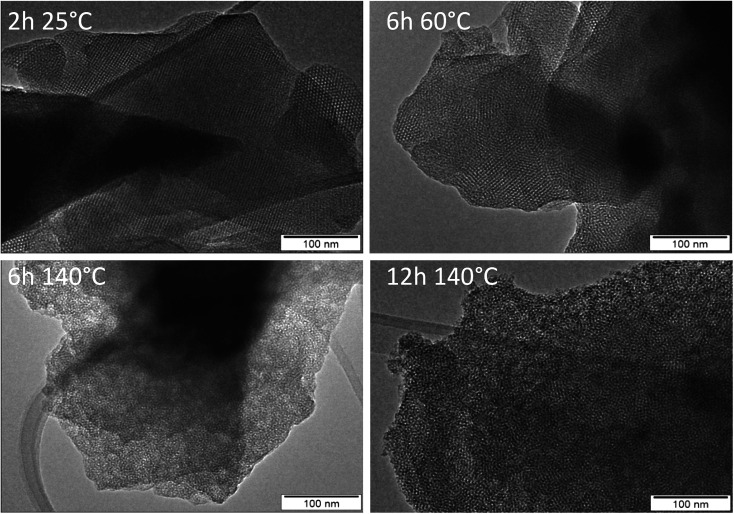
TEM images of materials after calcination of products obtained from AMS synthesis without aluminum as a function of synthesis time and temperature.

Finally, the development of the two different materials has been followed by transmission electron microscopy (TEM). The results for Al containing nano-beta and all silica AMS are summarized in Fig. 6 and 7 respectively. Fig. 6 illustrates the evolution of nano-beta, starting from an amorphous aluminosilicate at room temperature.

The TEM images suggest that some mesoscopic order of cylindrical micelles evolves during aging at 60 °C and at early stages of the hydrothermal treatment at 140 °C (Fig. S9†). With increasing reaction time, crystallization of nano-beta requires the formation of flat faces. Hence, cylindrical silicate shells break up to form zeolite beta particles (clearly visible after 5 and 14 h of hydrothermal reaction). The polar part of N_6_-diphe^6+^ is located within the zeolite micropores, the hydrophobic chains extend into the exterior of the crystallites and decorate their surfaces. The eventual sponge-like network of secondary mesopores is formed due to separation of ‘hydrophobic’ zeolite beta particles (external surface decorated with non-polar alkyl chains) and aqueous phase similar to a spinoidal decomposition, as it has been suggested in our previous study.^22^ The present study reveals that the presence of an aluminum source is essential for these processes to occur and that all-silica hierarchically porous zeolite nano-beta cannot be obtained in the studied conditions.

Without an aluminum source, AMS, an amorphous mesoporous silica, is obtained. The evolution of that material is illustrated by the TEM images in Fig. 7. The TEM image of cAMS (2 h, 25 °C) shows the mesostructure of the silica. The majority of the image shows cubic arrangement of the pores, *e.g.* view along the crystallographic [100] direction. At some places an apparent hexagonal arrangement is visible which is typical for a view along the [110] axis of a MCM-48-like silica with cubic *Ia*3̄*d* symmetry as shown by Terasaki, Ryoo and coworkers.^32,33^ Enlarged sections of the TEM images illustrating the different views are shown in Fig. S10.† The TEM data thus confirm the results of the low-angle XRD investigation, the mesostructure of cAMS shows cubic symmetry. The images also confirm that the mesoscopic order is lost during the hydrothermal reaction. An enlarged view of the TEM image of the AMS obtained after 12 h at 140 °C shows the highly disordered mesostructure of that material (Fig. S11†). Prolonged hydrothermal reaction also does not result in crystallization of zeolite beta if no aluminum is present in the reaction gel.

It should be noted that some larger spherical pores within the obtained materials support the assumption that N_6_-diphe forms spherical vesicles which we had made for the interpretation of SAXS data. Indications of such larger spherical voids are found in several materials obtained with and without aluminum in the reaction gel. Some examples are shown in Fig. S12.†

Considering the proposed eqn (1) it appears that the whole course of a hydrothermal synthesis very sensitively depends on seemingly small factors. The presence of an aluminum source in a synthesis gel for instance affects Δ*U*_attr_ and Δ*U*_rep_ due to an altered ionic strength at the initial stage of a zeolite synthesis. Since at that stage supramolecular recognition occurs, the starting point of the two investigated syntheses differs. Then, Δ*G*_solv_ and Δ*G*_lattice_ differ vastly at the later stages in the respective syntheses. The overall thermodynamic course of a hydrothermal synthesis (described by Δ*G*_h.t._syn_) producing zeolites can therefore be considered as a highly complex process that can be affected by small changes.

## Experimental

### Synthetic procedures

The reactants used in all these syntheses were tetraethylorthosilicate (TEOS, Sigma Aldrich, 98%), tetraethylammonium hydroxide (TEAOH, Sigma Aldrich, 35 wt%), and Al(*s*-OBu)_3_ (Sigma Aldrich, 97%). N_6_-diphe(Cl)_4_(Br)_2_ was synthesized according to a published procedure.^15^

The different investigated syntheses were performed as follows:

Aluminum-free material was obtained from a composition of 1SiO_2_ : 0Al_2_O_3_ : 0.03N_6_-diphe(Cl)_4_(Br)_2_ : 0.22Na_2_O : 53H_2_O.

Aluminum-containing zeolite nano-beta zeolite was obtained as described in previous studies^22^ with the molar ratios of 1SiO_2_ : 0.03Al_2_O_3_ : 0.03N_6_-diphe(Cl)_4_(Br)_2_ : 0.22Na_2_O : 53H_2_O. In our previous work that material was denoted as nano-beta_gel_, here we will refer to that zeolite as nano-beta for simplicity.

For both syntheses all reactants were premixed thoroughly and then TEOS was rapidly added as last component. Its hydrolysis was guaranteed by vortexing the mixture for 1 h. Then, the resulting thick gel was aged for 6 h at 60 °C. An N_6_-diphe/Si ratio of 0.2 was used. The aged gel was transferred into Teflon-lined stainless steel autoclaves and hydrothermal synthesis was performed at 140 °C for varying reaction times up to seven days under tumbling conditions (over-head tumbling). Yellowish solids were typically obtained as products. After calcination at 550 °C under air for 8 h, the final solids were obtained as white powders.

### Characterization

HR-SEM images were measured with a Hitachi S-5500 high-resolution scanning electron microscope with an acceleration voltage of 30 kV. Samples were placed on copper grids with 3 nm carbon coatings. Textural properties were studied with nitrogen sorption experiments on a Quantachrome Nova 3200e sorption analyzer. The samples have been outgassed and dehydrated at 623 K for 8 h under vacuum. Then nitrogen adsorption/desorption isotherms were measured at 77 K (liquid nitrogen). Calculation of pore size distributions and pore volumes was performed using the non-local density functional theory (NLDFT) method on the adsorption branches of the isotherms with models assuming sorption of nitrogen in cylindrical pores in oxidic materials at 77 K using the Quantachrome NovaWin and ASiQwin software packages. Total pore volumes were calculated from the volume adsorbed at relative pressure of *p*/*p*_0_ = 0.99, while micropore volumes were calculated with the *t*-plot method. The liquid-state NMR spectra were recorded at ^1^H Larmor frequency of 499.87 MHz on a Bruker Avance III 500 spectrometer equipped with a 5 mm BBFO+ probehead. All spectra were typically obtained with an rf pulse calibrated to a 90°-pulse of 10 μs. Samples were allowed to equilibrate at least 15 minutes at the given temperatures, calibrated to less than 0.5 °C. The 2D Nuclear Overhauser Enhancement Spectroscopy (NOESY) experiments were obtained with a standard Bruker gradient cross-relaxation pulse program “noesygpph” using a mixing time of 0.5 s. The indirect dimension was measured with 768 increments (8 scans per increment) with a recycling delay of 2 s. The indirect dimension was measured with 384 increments (4 scans per increment) with a recycling delay of 2 s. *R*_1_ and *R*_2_ relaxation experiments (inversion-recovery and Carr–Purcell–Meiboom–Gill pulse sequences) at varying temperature were typically obtained in pseudo-2D mode using 14 delay increments (from 0 to 10 s for *R*_1_, and 0 to 6 s for *R*_2_) and 8 scans per increment separated with a recycling delay of 10 s. Diffusion measurements were measured with a stimulated echo experiment with a diffusion time of 0.1 s and gradient delay of 1.2 ms. The experiment included 64 linear increments (4 scans per increment separated with a recycling delay of 5 s) of the bipolar gradient intensity corresponding to an attenuation of the signal from 100% to *ca.* 5%. All spectra were processed and analyzed in MNova 12. ^1^H MAS NMR spectra were recorded on a Bruker Avance 500 spectrometer at a resonance frequency of 500.1 MHz. The samples were packed in a 4 mm zirconia rotor and were spun at 10 kHz. The ^1^H chemical shifts are referenced to tetramethylsilane (TMS). XRD data were measured with a Stoe STADI P transmission diffractometer in Debye–Scherrer geometry. The instrument was equipped with a bent primary germanium monochromator allowing measurements with monochromatic CuK_α1_ radiation. Diffracted intensities were recorded with a position-sensitive detector (PSD) fabricated by Stoe. The PSD enables simultaneous recording of about 6° 2*θ*. Samples were measured in 0.7 mm borosilicate glass capillaries (wall thickness 0.01 mm) either in aqueous suspensions or as dry powders after separation of the nanoparticles by ultracentrifugation. Low-angle XRD patterns were recorded with line-collimated CuK_α_ radiation on a SAXSess mc^2^ instrument fabricated by Anton Paar. Scattered intensities were recorded with a Princeton Instruments CCD detector (SCX-TE 4300K/2), the samples were measured in 0.7 mm borosilicate glass capillaries (wall thickness 0.01 mm).

## Conclusions

Herein the structure directing effect of a surfactant-like template molecule that reportedly leads to hierarchical beta zeolites was investigated. Two cases are strikingly contrasting: gels with and without aluminum led to the formation of different materials. *In situ* and *ex situ* studies showed that the absence of an aluminum source leads to highly mesoporous materials (AMS), while the presence of aluminum warrants the formation of hierarchically porous nanozeolite beta. For the AMS materials it was found that a distinct precursor had already formed at ambient conditions with cubic arrangement of the pore system. Hydrothermal treatment did not lead to crystallization of zeolite beta, but substantial re-arrangement of the mesostructure resulting in a disordered mesoporous silica. In the presence of aluminum, the initial gel shows no significant mesoscopic order. Set into hydrothermal conditions, the aluminosilicate gel is gradually transformed into nano-beta with hierarchical pore structure. These two examples were used to illustrate the highly complex thermodynamic course of a zeolite hydrothermal synthesis that can be greatly influenced at many different levels.

## Conflicts of interest

There are no conflicts to declare.

## Supplementary Material

RA-010-D0RA03828H-s001

## References

[cit1] Groen J. C., Zhu W., Brouwer S., Huynink S. J., Kapteijn F., Moulijn J. A., Pérez-Ramírez J. (2007). J. Am. Chem. Soc..

[cit2] Hartmann M., Machoke A. G., Schwieger W. (2016). Chem. Soc. Rev..

[cit3] Sun M.-H., Huang S.-Z., Chen L.-H., Li Y., Yang X.-Y., Yuan Z.-Y., Su B.-L. (2016). Chem. Soc. Rev..

[cit4] Cejka J. , van Bekkum H. and Corma A., in Stud. Surf. Sci. Catal., Elsevier, 2007, vol. 168

[cit5] Beyer H. K. , in Molecular Sieves, vol. 3 Post-Synthesis Modifications, ed. H. G. Karge and J. Weitkamp, Springer, 2002, pp. 203–255

[cit6] Verboekend D., Vilé G., Pérez-Ramírez J. (2012). Cryst. Growth Des..

[cit7] Pavel C. C., Schmidt W. (2006). Chem. Commun..

[cit8] Eliášová P., Opanasenko M., Wheatley P. S., Shamzhy M., Mazur M., Nachtigall P., Roth W. J., Morris R. E., Čejka J. (2015). Chem. Soc. Rev..

[cit9] Kim J., Park W., Ryoo R. (2011). ACS Catal..

[cit10] Dorset D. L., Kennedy G. J., Strohmaier K. G., Diaz-Cabañas M. J., Rey F., Corma A. (2006). J. Am. Chem. Soc..

[cit11] Corma A., Rey F., Rius J., Sabater M. J., Valencia S. (2004). Nature.

[cit12] Choi M., Cho H. S., Srivastava R., Venkatesan C., Choi D.-H., Ryoo R. (2006). Nat. Mater..

[cit13] Cho K., Cho H. S., de Ménorval L.-C., Ryoo R. (2009). Chem. Mater..

[cit14] Na K., Choi M., Park W., Sakamoto Y., Terasaki O., Ryoo R. (2010). J. Am. Chem. Soc..

[cit15] Na K., Jo C., Kim J., Cho K., Jung J., Seo Y., Messinger R. J., Chmelka B. F., Ryoo R. (2011). Science.

[cit16] Gallego E. M., Portilla M. T., Paris C., León-Escamilla A., Boronat M., Moliner M., Corma A. (2017). Science.

[cit17] Piccione P. M., Laberty C., Yang S., Camblor M. A., Navrotsky A., Davis M. E. (2000). J. Phys. Chem. B.

[cit18] Rimer J. D., Trofymluk O., Lobo R. F., Navrotsky A., Vlachos D. G. (2008). J. Phys. Chem. C.

[cit19] Lupulescu A. I., Rimer J. D. (2014). Science.

[cit20] Grand J., Awala H., Mintova S. (2016). CrystEngComm.

[cit21] Castro M., Haouas M., Lim I., Bongard H. J., Schüth F., Taulelle F., Karlsson G., Alfredsson V., Breyneart E., Kirschhock C. E. A., Schmidt W. (2016). Chem.–Eur. J..

[cit22] Castro M., Losch P., Park W., Haouas M., Taulelle F., Loerbroks C., Brabants G., Breynaert E., Kirschhock C. E. A., Ryoo R., Schmidt W. (2018). Chem. Mater..

[cit23] Hould N., Haouas M., Nikolakis V., Taulelle F., Lobo R. (2012). Chem. Mater..

[cit24] Astorino E., Peri J. B., Willey R. J., Busca G. (1995). J. Catal..

[cit25] Stelzer J., Paulus M., Hunger M., Weitkamp J. (1998). Microporous Mesoporous Mater..

[cit26] Grünert A., Losch P., Ochoa-Hernández C., Schmidt W., Schüth F. (2018). Green Chem..

[cit27] Zana R., Lévy H., Danino D., Talmon Y., Kwetkat K. (1997). Langmuir.

[cit28] Swaddle T. W. (2001). Coord. Chem. Rev..

[cit29] Navrotsky A., Trofymluk O., Levchenko A. A. (2009). Chem. Rev..

[cit30] Bloembergen N., Purcell E. M., V Pound R. (1948). Phys. Rev..

[cit31] Haouas M., Petry P. D., Anderson W. M., Taulelle F. (2016). Inorganics.

[cit32] Kaneda M., Tsubakiyama T., Carlsson A., Sakamoto Y., Ohsuna T., Terasaki O., Joo S. H., Ryoo R. (2002). J. Phys. Chem. B.

[cit33] Carlsson A., Kaneda M., Sakamoto Y., Terasaki O., Ryoo R., Joo S. H. (1999). J. Electron Microsc..

