# Supplementary material for: Self-organization of silicates on different length scales exemplified by amorphous mesoporous silica and mesoporous zeolite beta using multiammonium surfactants
Source: RSC Adv. 2020 Jun 2;10(35):20928–38. doi: 10.1039/d0ra03828h (PMC9054314; doi:10.1039/d0ra03828h)
Supplement: RA-010-D0RA03828H-s001 [file RA-010-D0RA03828H-s001.pdf]

Electronic Supplementary Material (ESI) for RSC Advances.
This journal is © The Royal Society of Chemistry 2020

Self-Organization of Silicates on Different Length Scales Exemplified by Amorphous Mesoporous Silica and Mesoporous Zeolite Beta Using Multiammonium Surfactants

Maria Castro,[†] Pit Losch,[†] Christophe Farès,[†] Mohamed Haouas,[‡] Francis Taulelle,[§] Eric Breynaert,[§] Christine Kirschhock,[§] Woojin Park,[‖] Ryong Ryoo,[‖] Wolfgang Schmidt*[†]

[†] Max-Planck-Institut für Kohlenforschung, Germany

[‡] Institut Lavoisier de Versailles, UVSQ, CNRS, Université Paris-Saclay, Versailles, France

[§] Center for Surface Chemistry and Catalysis, KU Leuven, Belgium

[‖] CNCR, Institute for Basic Science, Republic of Korea

Supporting Information

Table S1. Textural properties of the different materials investigated.

Entry	Samples	Si/Al[a]	OSDA	S_{BET}[b] ($m^2.g^{-1}$)	V_t[c] ($cm^3.g^{-1}$)	V_{mic}[d] ($cm^3.g^{-1}$)	D_{meso}[e] (nm)
1	nano-Beta	15.4	N_6-diphe$(Cl)_4(Br)_2$	890	1.30	0.47	4.8
2	cAMS	∞	N_6-diphe$(Cl)_4(Br)_2$	1180	0.73	0.00	3.4
3	AMS	∞	N_6-diphe$(Cl)_4(Br)_2$	939	0.68	0.03	3.6

[a] The value is obtained by ICP analysis.
[b] S_{BET} is specific surface area calculated from N_2 adsorption data measured in p/p_0 range between 0.1 and 0.3 using the Brunauer-Emmett-Teller (BET) equation.
[c] V_t is total pore volume obtained at $p/p_0 = 0.95$.
[d] V_{mic} is micropore volume obtained via NLDFT analysis.
[e] D_{meso} is average mesopore diameter is obtained by NLDFT method (Quantachrome Autosorb software package) using NLDFT kernel for N_2 at 77 K on silica (cylindr. pore, NLDFT adsorption branch model).

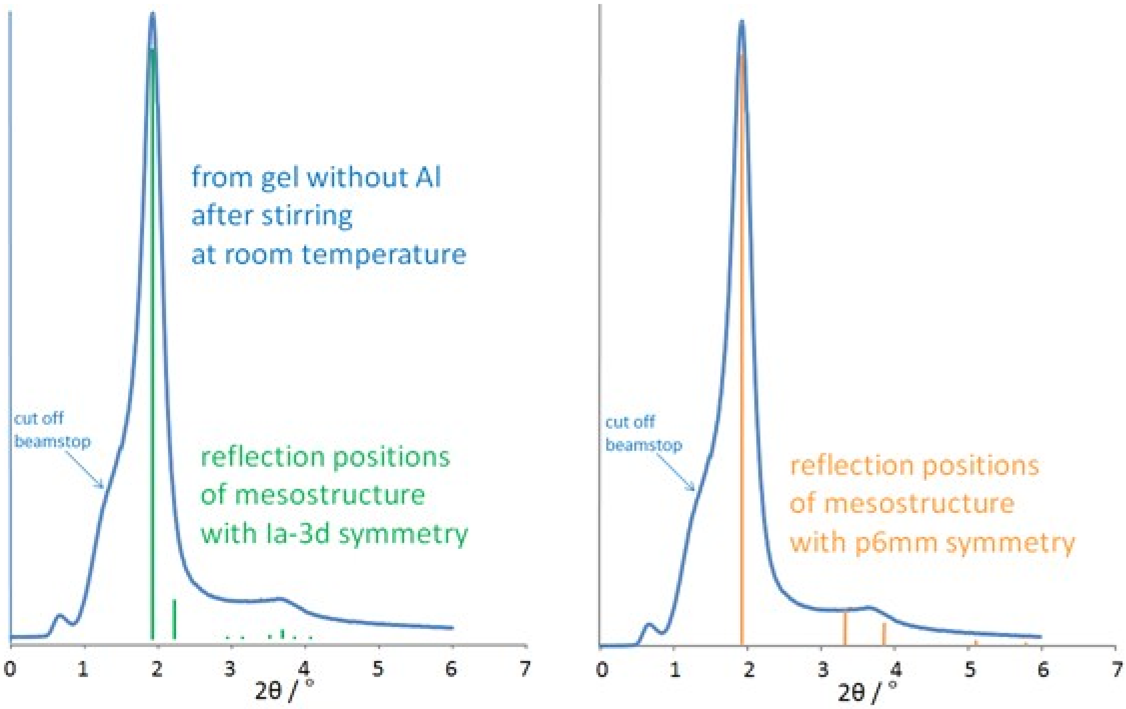

Figure S1. Low angle X-ray diffraction of AMS obtained after stirring at room temperature for 2 h. The intensities (green/orange lines) for Ia-3d (left) and p6mm (right) mesophases have been adapted from L.A. Solovyov, *Chem. Soc. Rev. 42* (2013) 3708-3720 and L.A. Solovyov, O.V. Belousov, R.E. Dinnebier, A.N. Shmakov, S.D. Kirik, *J. Phys. Chem. B*, *109* (2005) 3233-3237. The positions of the lines representing the two symmetries have been calculated for the respective d value of the strongest reflection. The diffraction patterns fit best to the Ia-3d symmetry (MCM-48 like) and not to the hexagonal p6mm symmetry (MCM-41 like).

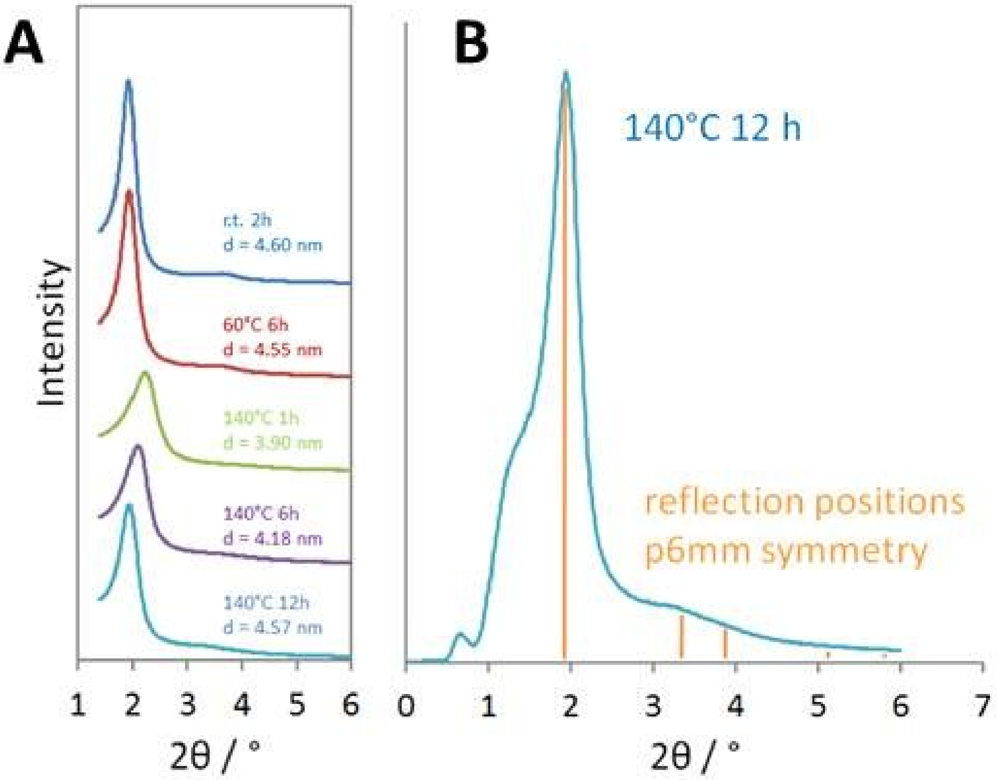

Figure S2. A) Low angle X-ray diffraction of AMS obtained after stirring at room temperature for 2 h, aging at 60°C for 6h, and hydrothermal reaction at 140°C for 1, 6, and 12 h. The materials had been calcined at 140°C for 4 h. B) XRD pattern of calcined sample obtained after reaction at 140°C for 12 h with reflection positions expected for hexagonal p6mm symmetry.

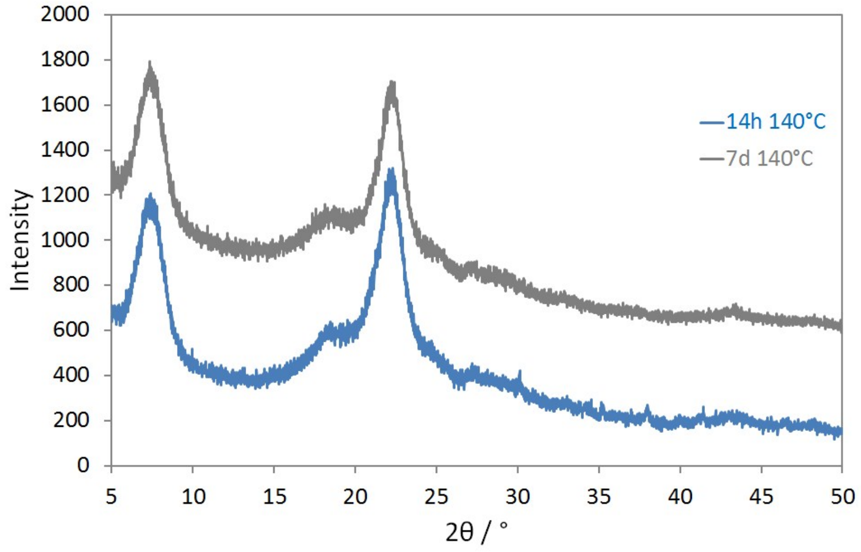

Figure S3. XRD patterns of nano-Beta after 14 h and 7 d of hydrothermal reaction at 140°C.

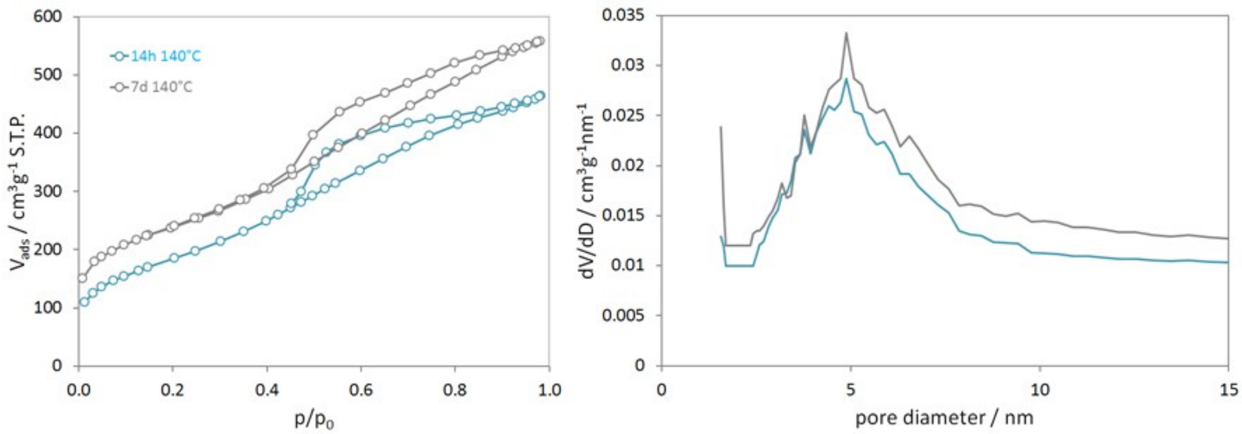

Figure S4. a) Nitrogen sorption isotherms and b) Pore size distributions (calculated with NLDFT from adsorption branches of the isotherms) of nano-Beta after 14 h and 7 d of hydrothermal reaction at 140°C.

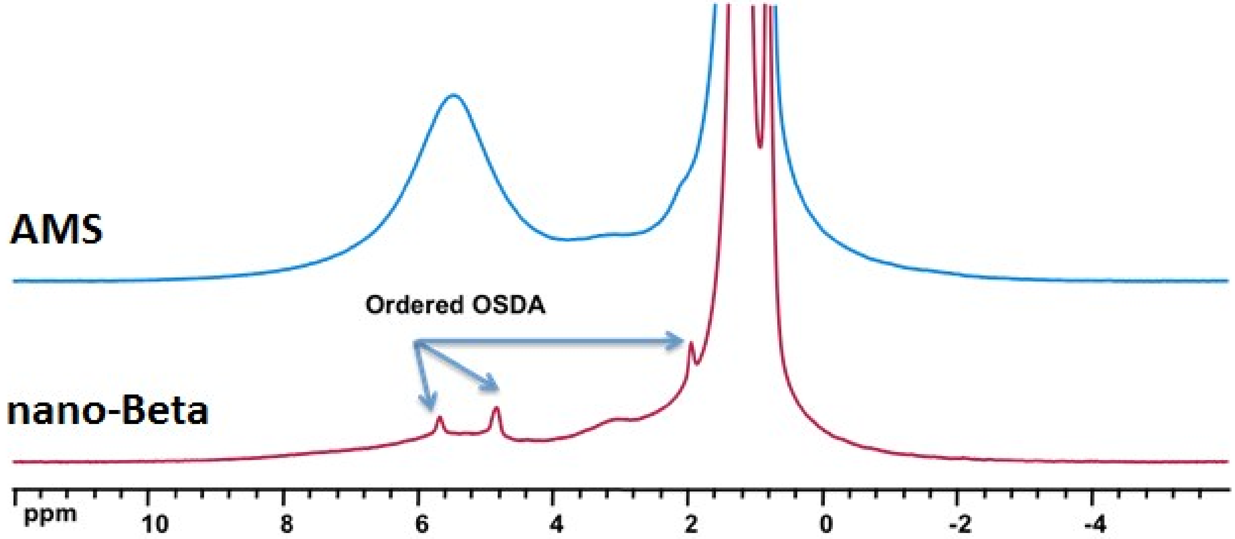

Figure S5. ^{1}H-MAS-NMR of the two different final materials obtained in absence (AMS) and presence of aluminum source (nano-Beta).

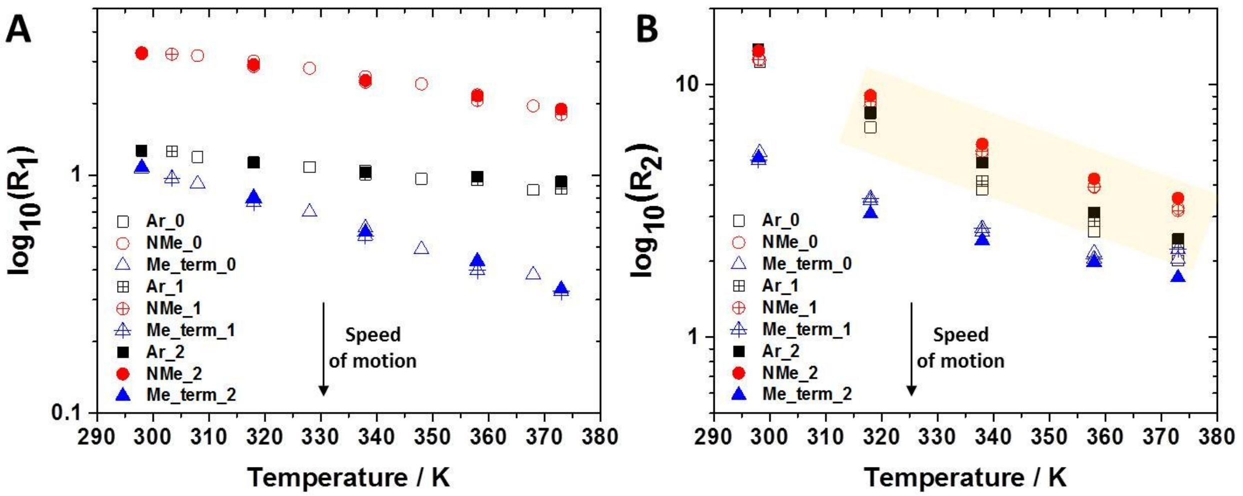

Figure S6. Relaxation rates (R_1 and R_2) data for the different regions of the template molecule at different temperatures and different stages of the synthesis: _0 free template; _1 template with Al source; _2 template with Al and Si source. **A**) Logarithm of R_1 values are plotted as a function of temperature, **B**) Logarithm of R_2 values are plotted as a function of temperature, here significant changes are observed for the NMe and aromatic groups upon addition of Al and Si sources at temperatures relevant to the synthesis.

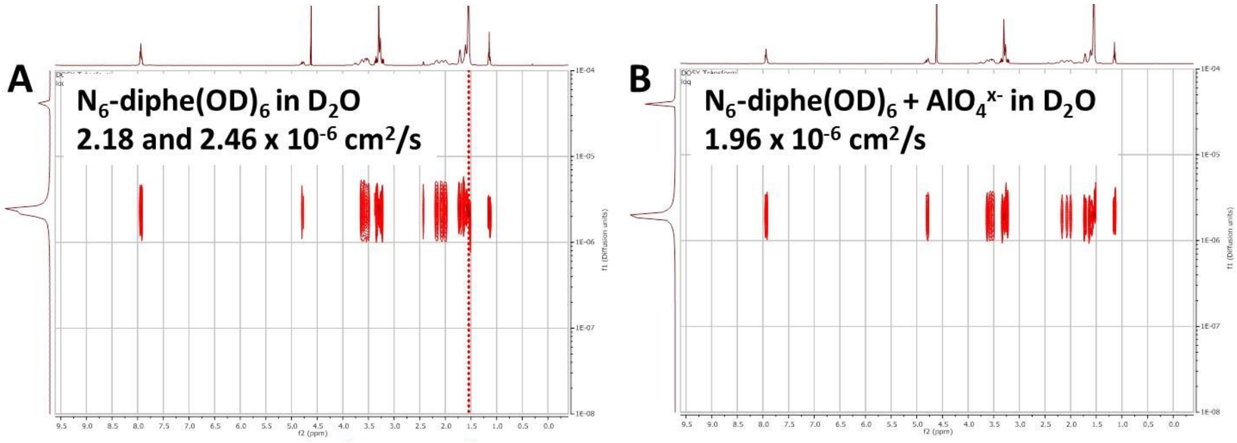

Figure S7. DOSY spectra of A) N_6-diphe and B) N_6-diphe in the presence of aluminum.

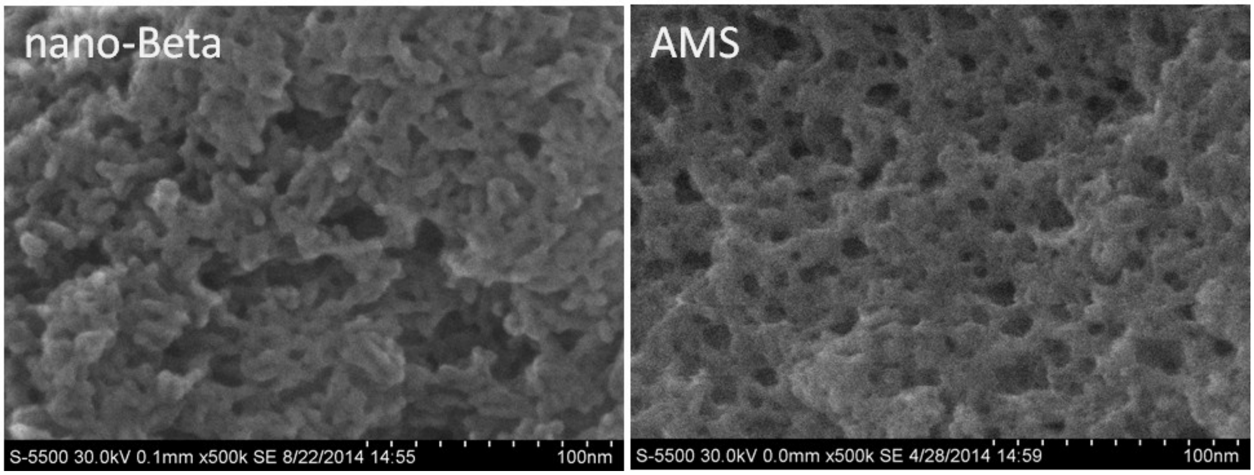

Figure S8. SEM micrographs of nano-Beta consisting of Beta nano-rods (left) and holey sponge-like structure of AMS.

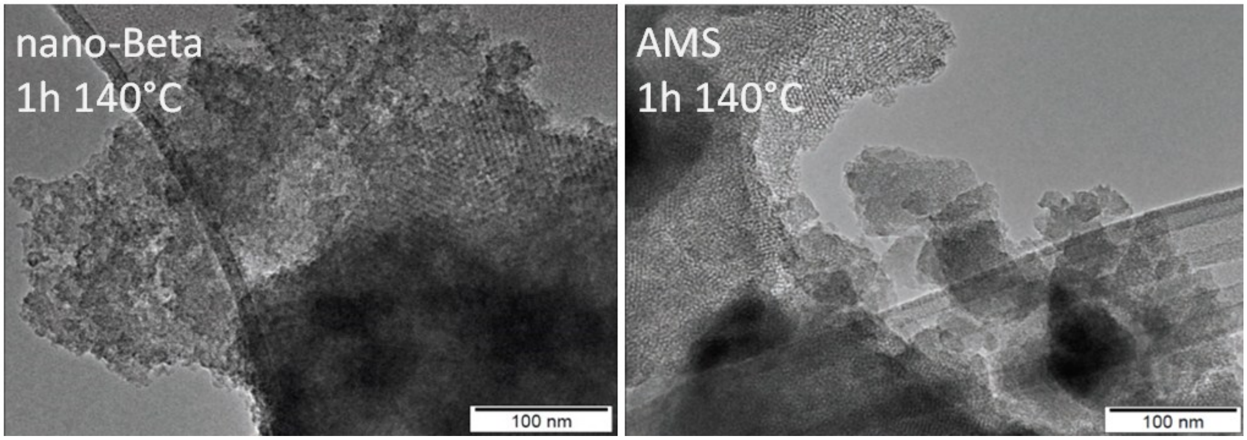

Figure S9. TEM images of the dried and calcined reaction gel of nano-Beta and AMS syntheses after 1 h at 140°C.

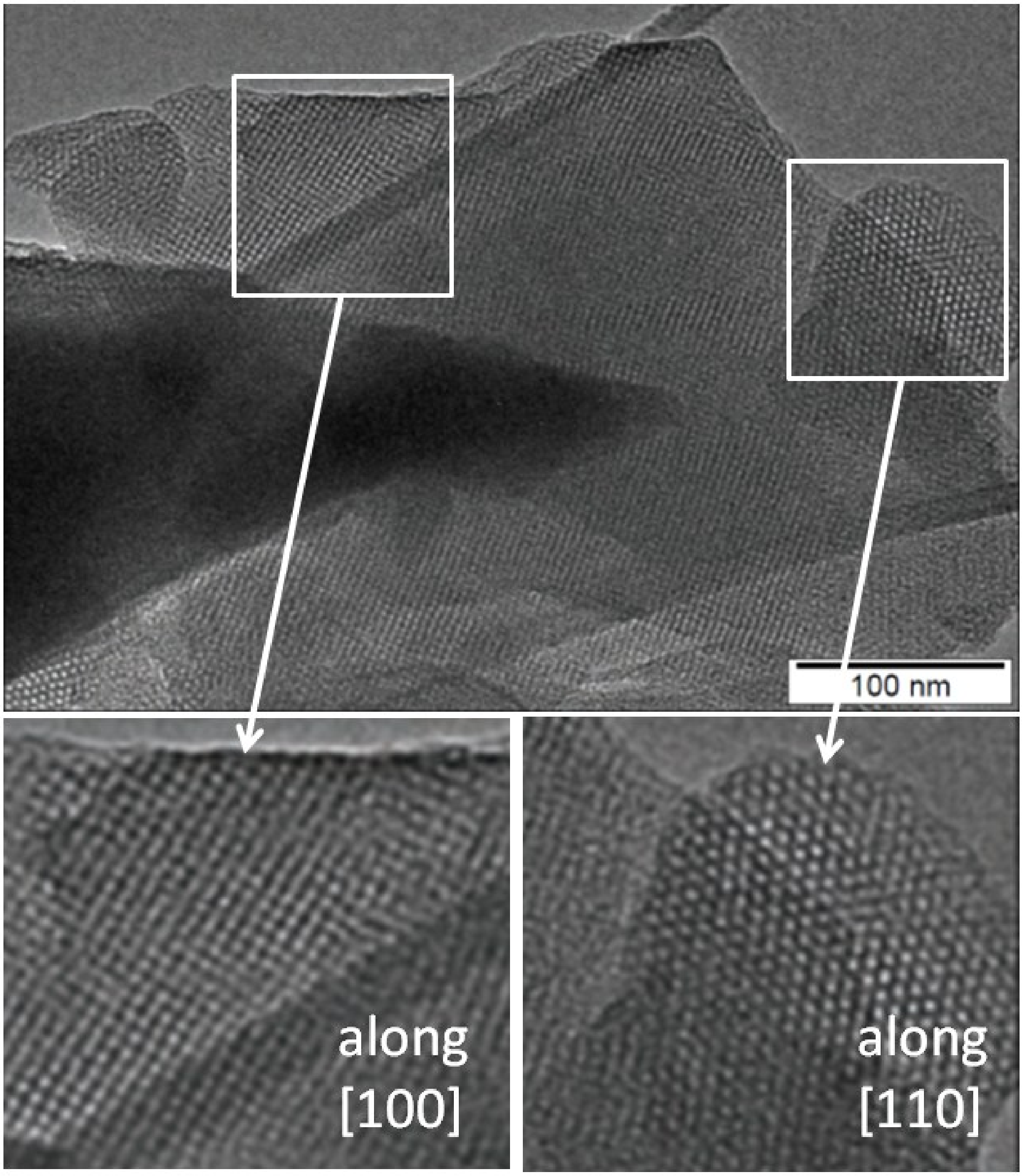

Figure S10. Enlarged sections of the TEM image of cAMS (obtained after stirring of reaction gel without Al for 2 h at 25°C) with cubic Ia-3d symmetry. The enlarged images show views along the crystallographic [100] and the [110] directions.

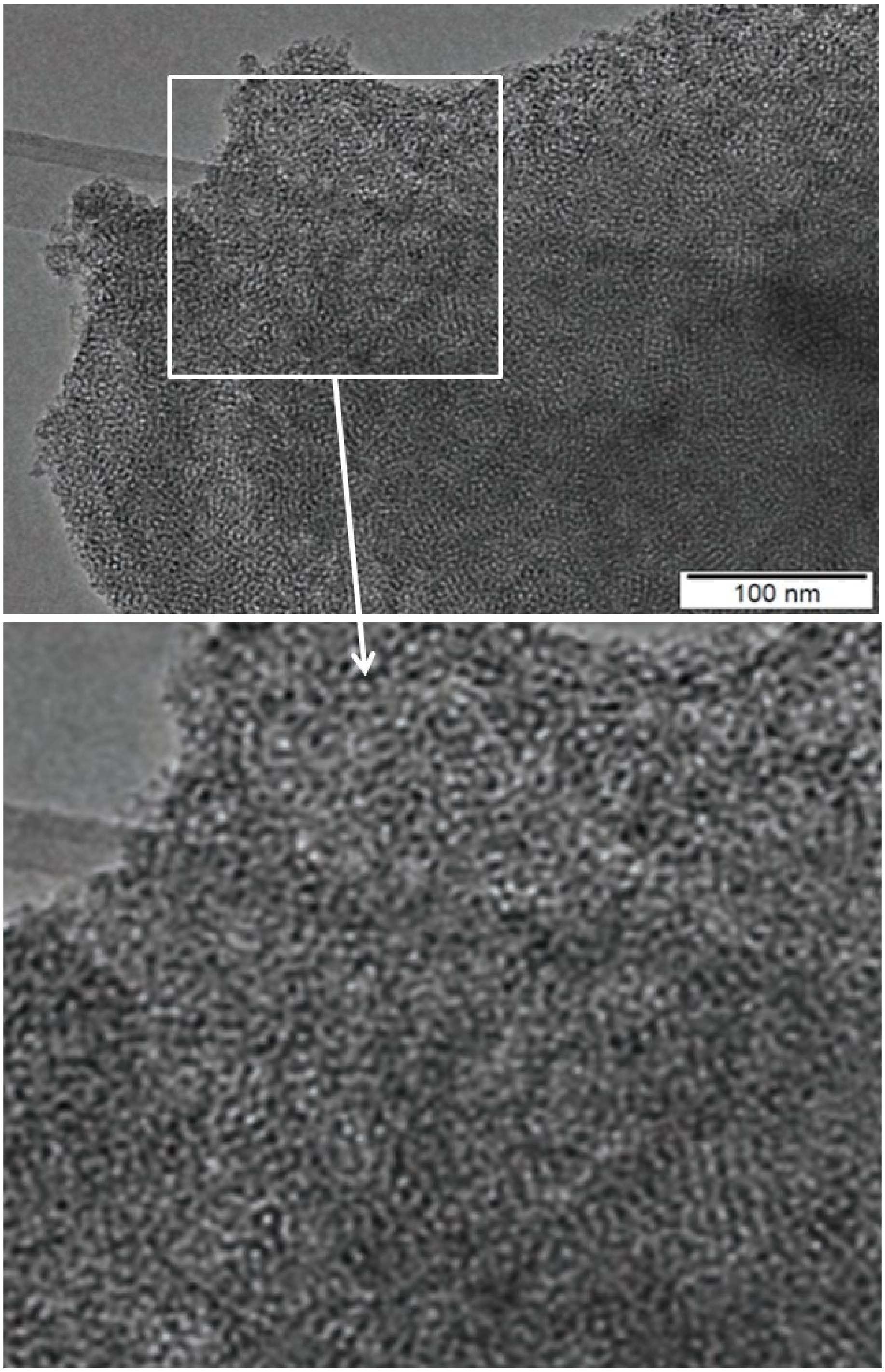

Figure S11. Enlarged section of the TEM image of AMS (obtained after hydrothermal reaction for 12 h at 140°C). The enlarged image shows a highly disordered mesostructure.

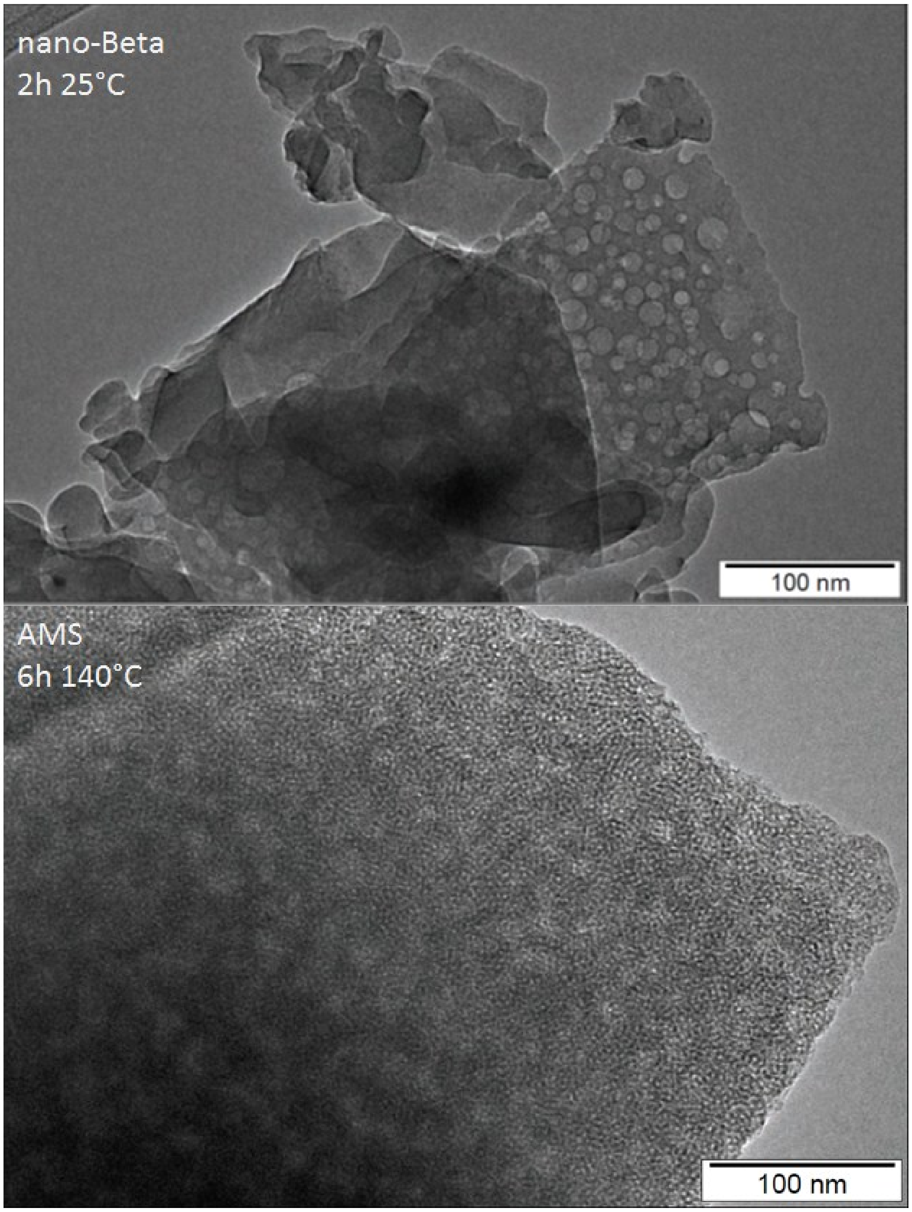

Figure S12. TEM images of the dried and calcined reaction gels of the nano-Beta synthesis after mixing for 2 h at room temperature (top) and AMS after 1 h at 140°C. The images show indications for spherical voids supporting the existence of multilayered vesicles (containing large amounts of N_6-diphe) in reaction gel prior to calcination.
